# Challenges of dermoscopic assessment of basal cell carcinoma on tattooed skin

**DOI:** 10.1016/j.jdcr.2026.01.058

**Published:** 2026-02-07

**Authors:** Joana Margarida Reis, José Carlos Cardoso, André Oliveira

**Affiliations:** a Department of Dermatology and Venereology, Hospital Garcia de Orta, Almada, Portugal; b Dermatology Department - Unidade Local de Saúde de Coimbra, Coimbra, Portugal; c Skin Screening - Dermatology Unit, Champalimaud Clinical Centre - Champalimaud Foundation, Lisboa, Portugal

**Keywords:** arborizing vessels, basal cell carcinoma, corkscrew vessels, dermoscopy, dotted vessels, erosions, hairpin vessels, irregular linear vessels, shiny white structures, tattoo, vascular polymorphism

## Introduction

Tattoos are becoming increasingly popular as a form of body art, with prevalence estimates of 35% to 48% in Europe.^1^ Although infectious and allergic complications are frequent, malignant tumors have been less described.^2^^,^^3^ This is remarkable in basal cell carcinoma (BCC), because its high incidence contrasts with rare literature reports occurring on tattooed skin. Diagnosing skin tumors in tattooed skin can be challenging, as exogenous pigment may mask or mimic key structures, complicating clinical examination and dermoscopic interpretation.^4^^,^^5^ In our series, all BCCs were associated with tattoo pigment, which was histologically identifiable in every case. We present a series of 5 tattoo-associated BCCs, emphasizing their dermoscopic features. Clinical and histological characteristics of tattoo-associated BCC are represented in Table I and dermoscopic features are described in Table II.

**Table I:** Clinical and histologic characteristics of tattoo associated BCC

Case nr	Age/sex	Fototype	Tattoo duration (yr)	Tatto location	Pigment color	Dimension (mm)	Evolution time (mo)	Lesion type	Histopathology
1	45/F	II	15	Scapular region	Black	7	12	Plaque	Superficial BCC
2	47/F	I	2	Thigh	Black	7	6	Papule	Nodular BCC
3	55/M	I	17	Chest	Black	5	Unknown	Papule	Nodular BCC
4	64/M	II	20	Arm	Red	17	24	Plaque	Nodular BCC
5	58/F	I	18	Shoulder	Black	7	12	Papule	Nodular BCC

Clinical and histologic features of the 5 BCCs arising in tattooed skin: 5 patients (3 women), aged between 45 and 64 years; tattoo duration ranged from 2 to 20 years, and clinical evolution from 6 to 24 months. Histopathology showed 4 nodular BCCs and 1 superficial.

**Table II:** Dermoscopic features of tattoo-associated BCC

Case nr	Vascular structures						Pigmented structures	Shiny white structures
	Arborizing vessels	Irregular linear vessels	Corkscrew vessels	Hairpin vessels	Dotted vessels	Vascular polymorphism		
1	-	+	-	-	+	+	Gray ovoid nests	+
2	-	+	+	-	-	+	-	+
3	-	+	-	-	+	+	-	-
4	-	+	-	-	+	+	-	+
5	-	+	-	+	+	+	-	+

Pigmented dermoscopic criteria were frequently obscured by exogenous tattoo pigment, whereas vascular patterns and shiny white structures remained consistently identifiable.

*+*, Present; *-*, absent.

## Case 1

**Fig 1:**
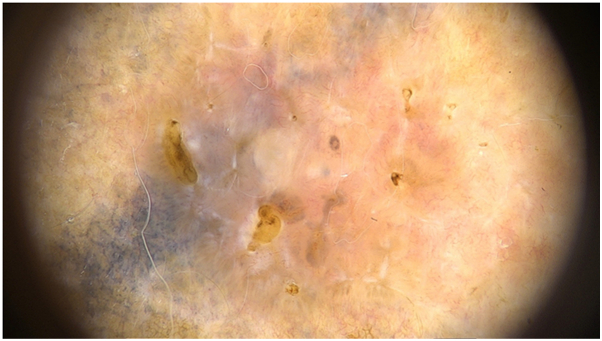
Dermoscopy of BCC revealing irregular linear and dotted vessels. Shiny white structures, concentric structures, gray ovoid nests and erosions are also seen.

A 45-year-old woman with a 15-year-old tattoo presented with an associated lesion on the scapular area with 12 months of evolution. Dermoscopy revealed vascular polymorphism with irregular linear and dotted vessels. Shiny white structures, large globules, grey ovoid nests and erosions were also observed (Fig 1). Histopathology confirmed superficial BCC.

## Case 2

**Fig 2:**
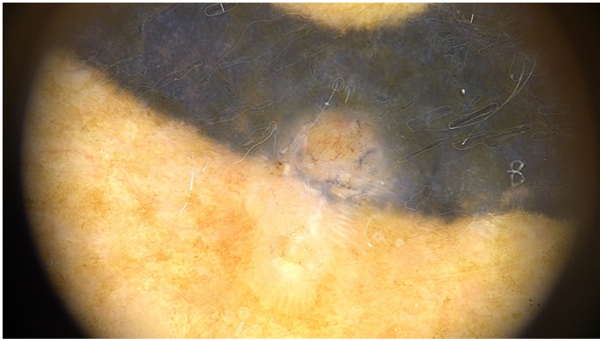
BCC revealing irregular linear vessels, corkscrew vessels, and shiny white structures.

A 47-year-old woman with a 2-year-old tattoo developed an associated lesion on the thigh with a 6 month history of growth. Dermoscopy showed vascular polymorphism with irregular linear and corkscrew vessels. Shiny white structures were also present (Fig 2). Histopathology revealed nodular BCC.

## Case 3

**Fig 3:**
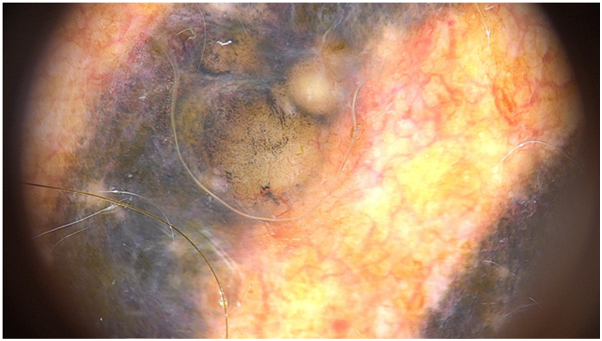
BCC dermoscopy with evident vascular polymorphism, with irregular linear and dotted vessels.

A 55-year-old man with a 17-year-old tattoo presented with a lesion on the chest of uncertain duration. Dermoscopy revealed vascular polymorphism with irregular linear and dotted vessels (Fig 3). Histopathology confirmed nodular BCC.

## Case 4

**Fig 4:**
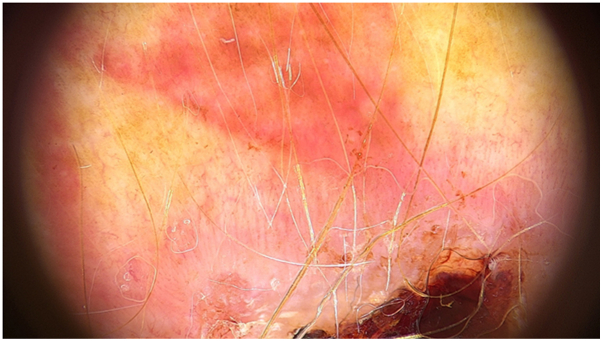
BCC dermoscopy revealing vascular polymorphism, with irregular linear vessels and dotted vessels. Ulceration and shiny white structures are also present.

A 64-year-old man with a 20-year-old tattoo presented with a concomitant lesion on his arm with 2 years of evolution. Dermoscopy demonstrated vascular polymorphism with irregular linear and dotted vessels. Ulceration and shiny white structures were also present (Fig 4). Histopathology confirmed nodular BCC.

## Case 5

**Fig 5:**
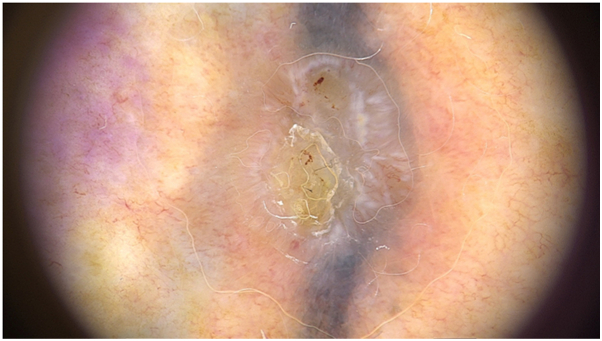
BCC dermoscopy with vascular polymorphism, with irregular linear vessels, hairpin and dotted vessels. Shiny white structures and erosions are also seen.

A 58-year-old woman with an 18-year-old tattoo presented with a lesion on her shoulder with a 12 months of evolution. Dermoscopy revealed vascular polymorphism with irregular linear, dotted, hairpin, and corkscrew vessels. Shiny white structures and erosions were also present (Fig 5). Histopathology confirmed nodular BCC.

## Discussion

Forty-one cases of BCC on tattooed skin have been reported to date,^1^ yet only 3 included dermoscopic descriptions.^6^^,^^7^ Our series suggests that classical pigmented dermoscopic criteria of basal cell carcinoma, such as ovoid nests, spoke-wheel structures, and maple-leaf-like areas may be obscured by exogenous tattoo pigment. This masking effect can reduce the sensitivity of dermoscopy and contribute to diagnostic challenges. Otherwise, in all cases, vascular structures were readily identifiable, with a predominance of polymorphous vessels, including linear-irregular vessels, despite the consistent absence of arborizing vessels typically associated with non-ulcerated nodular BCC. These vascular patterns, usually seen in amelanotic or hypomelanotic melanoma and aggressive fast-growing palpable tumors, may reflect optical artifacts induced by tattoo ink, as well as local inflammatory or elastotic changes. In addition, shiny white structures, another key dermoscopic feature of BCC, appear to maintain diagnostic relevance across most cases. Taken together, these findings support a dermoscopy-first approach focused on vascular morphology and shiny white structures when evaluating suspicious lesions on tattooed skin, rather than classical pigmented criteria. Our findings are intended to be descriptive and to emphasize practical diagnostic considerations when evaluating suspicious lesions on tattooed skin. Careful and systematic dermoscopic assessment of tattooed skin therefore remains essential.

## Conflicts of interest

None disclosed.
